# A phytochemical comparison of saw palmetto products using gas chromatography and ^1^H nuclear magnetic resonance spectroscopy metabolomic profiling

**DOI:** 10.1111/jphp.12198

**Published:** 2014-01-13

**Authors:** Anthony Booker, Andy Suter, Ana Krnjic, Brigitte Strassel, Mire Zloh, Mazlina Said, Michael Heinrich

**Affiliations:** a Centre for Pharmacognosy and Phytotherapy, University of LondonLondon, UK; c UCL School of Pharmacy, University of LondonLondon, UK; b A.Vogel, Bioforce AGRoggwil, Switzerland; d Department of Pharmaceutics and Industrial Pharmacy, Faculty of Pharmacy, King Abdulaziz UniversityJeddah, Saudi Arabia

**Keywords:** fatty acids, gas chromatography, ^1^H nuclear magnetic resonance spectroscopy, metabolomics, saw palmetto

## Abstract

**Objectives:**

Preparations containing saw palmetto berries are used in the treatment of benign prostatic hyperplasia (BPH). There are many products on the market, and relatively little is known about their chemical variability and specifically the composition and quality of different saw palmetto products notwithstanding that in 2000, an international consultation paper from the major urological associations from the five continents on treatments for BPH demanded further research on this topic. Here, we compare two analytical approaches and characterise 57 different saw palmetto products.

**Methods:**

An established method – gas chromatography – was used for the quantification of nine fatty acids, while a novel approach of metabolomic profiling using ^1^H nuclear magnetic resonance (NMR) spectroscopy was used as a fingerprinting tool to assess the overall composition of the extracts.

**Key findings:**

The phytochemical analysis determining the fatty acids showed a high level of heterogeneity of the different products in the total amount and of nine single fatty acids. A robust and reproducible ^1^H NMR spectroscopy method was established, and the results showed that it was possible to statistically differentiate between saw palmetto products that had been extracted under different conditions but not between products that used a similar extraction method. Principal component analysis was able to determine those products that had significantly different metabolites.

**Conclusions:**

The metabolomic approach developed offers novel opportunities for quality control along the value chain of saw palmetto and needs to be followed further, as with this method, the complexity of a herbal extract can be better assessed than with the analysis of a single group of constituents.

## Introduction

Saw palmetto (*Serenoa repens* (W.Bartram) Small (Arecaceae.syn.: *Sabal serrulata*(Michx.) Schult.f.)) is a small, low-growing, dwarf-palm tree, native to South-eastern North America, particularly Florida. The American Indians of this region used the berries medicinally and as a food source, long before the arrival of the Europeans. Preparations of saw palmetto are made of the ripe, partially dried olive size berries, and they were used traditionally for a wide range of indication like bronchitis, common colds, for the treatment of genito-urinary disorders of men and women, and as a sexual stimulant. In the United States, the plant gained recognition as an official remedy in 1906 being first mentioned in the US Pharmacopoeia,[1] followed in 1926 with the inclusion in the US National Formulary.[2]

Since the 1990s, saw palmetto has been one of the top 10 top-selling herbal medicines in the United States, and the worldwide turnover of *Serenoa* preparations is likely to be 700 million dollars per annum.[3] In Europe, the extract of saw palmetto is the herbal medicine most frequently used for the treatment of the urological symptoms caused by benign prostatic hyperplasia (BPH), and the evidence from over 35 clinical trials supports the use of standardised saw palmetto extract for the treatment of lower urinary symptoms associated with BPH. Treatment with the extract has shown increased peak urine flow, and decreased nocturia, without increasing serum prostate-specific antigen (PSA) levels.[4] In a pilot trial, we could show that saw palmetto also had a positive effect on concomitant sexual dysfunctions in BPH patients.[5] The dose recommended for a lipophilic saw palmetto extract is 320 mg daily administered once daily. Usually, the extract contains 70–95% free fatty acids like capric, caproic, caprylic, lauric, myristic, oleic, linoleic, linolenic, stearic and palmitic acids[6] with their ethyl esters and glycerides that occur most likely during the extraction process. Further phytosterols like β-sitosterol and β-sitosterol 3-O-D-glucoside, campesterol, and stigmasterol[7] are found with about 0.1% of the total mass of the berries. Other constituents are carbohydrates such as mannitol and polysaccharides with galactose and arabinose, triterpenes, aromatic acids like ferulic and vanillic acid, β-carotens, the vitamine E derivates γ-tocopherol and δ-tocopherols,[6] mono-acyl glycerides like 1-monolaurin and 1-monomyristin,[8] and the monoamine tyramine.[9]

A combination of several factors has been implicated as potential mode of action for saw palmetto. The main properties to consider are its anti-androgenic, pro-apoptotic and anti-inflammatory effects.[10] Fatty acids are considered to be key active constituents, and Bayne *et al*. reported its effectiveness as a dual inhibitor of 5α-reductase activity in the prostate gland.[11]

There are many extracts on the market obtained using different extraction processes resulting in a lack of equivalence of these products. One study examining the content of saw palmetto in six commercial preparations compared with the amounts as stated on labels reported a −97% to +140% difference compared with the label claim. Half of the samples tested contained less than 25% of the stated amount.[12] Furthermore, Habib and Wyllie commented that the potency of tested extracts appeared to be significantly different with respect to their isoenzyme inhibition and suggested that only extracts with evidence of activity and clinical efficiency should be considered for the treatment of BPH.[13] The only official quality marker so far for saw palmetto preparations is to be found in the European Pharmacopoeia that requires lauric acid to be at least 20% of the total fatty acid.[14]

To gain a better understanding of the chemical variability of these products, we analysed 57 different products from nine countries that contain saw palmetto as monopreparations or as a part of multicomponent supplements. All test products are used for treatment of BPH symptoms and were analysed either with gas chromatography or ^1^H nuclear magnetic resonance (NMR) spectroscopy with subsequent principal component analysis (PCA).

Consequently, the goal of this novel comparative study was to evaluate the quality of saw palmetto preparations by using fatty acids as quality markers and, for the first time, assess if ^1^H NMR spectroscopy and PCA are suitable, robust and fast methods for quality evaluation of these products.

### Metabolomics and principal component analysis

Metabolomics is the term used for the comprehensive, non-biased, high-throughput analyses of complex metabolite mixtures such as those typically seen in plant extracts. Achieving a broad overview of metabolic composition is very complex and requires the establishment of a fully integrated approach for the optimisation of sample extraction, metabolite separation/detection/identification, automated data gathering, processing and analysis, and quantification.[15] Metabolomics has become a well-recognised method for the study of all types of organisms and complements the data obtained by the other ‘omics’-technologies: genomics, transcriptomics and proteomics.[16] One of the main problems associated with metabolomics is that the metabolome consists of a wide range of compounds at very different concentrations and with different polarities. At present, there is no single solvent capable of solubilising the whole range of chemical constituents. The choice of extraction solvent is thus limiting the view on the metabolome. In general, metabolomic studies should be designed to detect as many metabolites as possible in an organism[17] In this study, plant extracts are the focus and so the metabolomic profile of the plant will have already been simplified. As this study is concerned with the analysis and quality of herbal medicinal products, this simplification fits in with the aims and objectives of the study, however, if we chose to look at the saw palmetto plant, the extraction steps would need to be optimised to attempt to extract the full range of metabolites.

NMR is an effective tool for the quality control of medicinal plants or herbal medicinal products[18] The advantages of NMR spectroscopy over other techniques such as mass spectroscopy (MS) for metabolomics applications include the relative ease of sample preparation, non-destructive analysis, potential to identify a broad range of compounds, enhanced capacity for definitive chemical compound identification, and provision of structural information for unknown entities.[19] ^1^H NMR spectroscopy is an ideal tool for large-scale plant metabolomics data collection. Multivariate or pattern recognition techniques such as PCA are valuable techniques for the analysis of data obtained by NMR spectroscopy. In combination with PCA, NMR spectroscopy has been applied to the metabolomics profiling of plants and herbal medicines.[20]

PCA is a math-based method of reorganising information found in a data set of samples. It can be used when the set contains information from only a few variables, but it is most useful when there are large numbers of variables, as in spectroscopic data. What PCA does is to discover new variables, called ‘principal components’, which account for the main variability of the data.[21] Data are represented in ‘*n*’ dimensional space, where *n* is the number of variables, and is reduced into a few principal components, which are descriptive dimensions that describe the maximum variation within the data. The principal components can be displayed in a graphical fashion as a ‘scores’ plot. This plot is useful for observing any groupings in the data set and, in addition, will highlight outliers that may be due to errors in sample preparation or instrumentation parameters. PCA models are constructed using all the samples in the study. Coefficients by which the original variables must be multiplied to obtain the PC are called ‘loadings’. The numerical value of a loading of a given variable on a PC shows how much the variable has in common with that component. Thus, for NMR data, ‘loading plots’ can be used to detect the spectral areas (metabolites) responsible for the separation in the data.[22]

## Materials and Methods

### Test samples

Fifty-seven samples were obtained from retail outlets or pharmacies from Canada, Finland, Germany, the Netherlands, the United Kingdom, South Korea, Spain, Switzerland and the United States. Of these products that were soft gel or hard gel capsules, tablets or tinctures, 29 were monopreparations containing only saw palmetto, and 28 were combined with other constituents such as vitamins, herbal extracts or minerals (labelled ‘combi-preparations’). Thirty-four products were tested with ^1^H NMR spectroscopy, 46 with gas chromatography and 26 preparations in both analyses. For the determination of the fatty acids with gas chromatography, 19 mono and 27 combi were analysed. The extracts were in the majority not well-declared on the packages or package leaflets as they were sold as food supplements and were not registered, so only for 12 products proper drug- solvent (extractant) ratio and the extractant were available. The declarations given showed that the majority of the saw palmetto extracts were lipophilic, at least one (SP14) contained also crude saw palmetto berry powder. A detailed description of all the test items including which analysis, gas chromatography (GC) or NMR, was carried out with which product is given as supplementary material (published online).

### Determination of fatty acids with gas chromatography

The method is based on the esterification and subsequent separation and analysis using gas chromatography calculated against heptadecanic acid as internal standard. This is a modified method of the German Society for Fat Science for fatty acid methyl esters, where in the end, the free fatty acids are determined.[23]

For each preparation, 1 g of the capsule content or of the tablets was extracted for 45 min with 70-ml hexane together with 5-ml heptadecanic acid. The extraction solution was concentrated to ca. 25 ml on the rotary evaporator and dried with sodium sulfate.

Six millilitres of 2% methanolic sodium hydroxide solution were added to the residue and heated to the boiling point for 10 min, then 5-ml boron trifluoride-methanol complex were added and the whole mixture was boiled for another 2 min. Finally, 20-ml heptane were added, stirred and the mixture cooled down.

Afterwards, saturated sodium chloride solution was added until the heptane phase ascended into the flask neck. About 1 ml of the resulting heptane phase was dried in a centrifuge tube with water-free sodium sulfate; the clear yellowish green solution was used for the GC-flame ionisation detector. The analysis was performed twice for each test preparation with a gas chromatograph (Trace GC ultra, Thermo Electron Corporation, Waltham, MA, USA) using an Optima 5 capillary column containing 5% Phenyl and 95% Methylpolysiloxan, 25 m × 0.32 mm iD with 0.25 μm layer thickness.

The column was held isothermally for 1 min at 100°C, then the temperature was programmed to increase at a rate of 5°C/min to 190°C where it was kept for 5 min. The injector was set to 250°C, and an amount of 1-μl solution was injected using the split mode with hydrogen as carrier gas at a constant pressure of 40 kPa.

The system suitability was given when in the chromatogram, the resolution between the main peak lauric acid and myristic acid amounted to at last 10. The single fatty acids that were assigned according to the retention time of the peaks and were calculated in reference to the area of heptadecanoic acid. The areas were integrated by using Chromcard software. The methods precision is 0.9%, and the correlation in linearity is 0.998.

### ^1^H NMR spectroscopy

#### Solvents, reagents and chemicals

Deuterated methanol-d4 (99.8%) lot no. 10C-522, Tetramethylsilane (99.9%) lot no. 81–140 and deuterated chloroform-d with added 0.05% v/v Tetramethylsilane lot no. 9F-330 were purchased from Cambridge Isotope Laboratories, Inc., Andover, MA, USA.

Reference standards: Caproic acid ethyl ester, lot no. S28172-020; oleic acid, lot no. 098 K5216, and stigmasterol, lot no. 53F0322 were purchased from Sigma-Aldrich Chemicals, St Louis, MO, USA.

#### Apparatus and instrumentation

Bruker Avance NMR Spectrophotometer (500 MHz) with Topspin software version 1.3
AMIX Bruker Biospin multivariate analysis software version 3.0
Soft Independent Modeling of Class Analogy Software (SIMCA) Version 13.0
Ependorff Minispin plus centrifuge, model 5453, Serial no. 0031564
Fisher brand ultrasound bath, model D-78224, serial no. 004472044
Rotamixer, rotary mixer, serial no. 8011
Gilson micro-pipettes 200ul, serial no.AC55298 and 1000ul, serial no. AD63010 (calibrated 15.6.10)
Reaction tubes, 1.5 ml eppendorf supplied by Griener Bio-One, Germany
Wilmad LabGlass NMR sample tubes, 5 mm economy, 7″Length, 100 MHz

#### Sample preparation

The method for the extraction of plant samples was developed from a method described by Frederich et al.[24] Initial experiments were carried out using deuterated methanol-d4, but it was found that many of the soft gel capsule extracts did not dissolve satisfactorily in deuterated methanol-d4, and so the solvent was changed to deuterated chloroform-d containing 0.05% tetramethylsilane (TMS). The solubility of the saw palmetto soft gel capsule extract products was improved using this solvent, and so two experiments were carried out. Deuterated chloroform-d was identified as the solvent of choice for the ^1^H NMR spectroscopy analysis of soft gel capsule extracts, and deuterated methanol-d4 was used for the crude powders and powder extracts.

Methanol soluble: Ten brands of single ingredient crude powder, powder extract or tablet, and four brands of multicomponent powders were tested.

Chloroform soluble: Thirteen brands of single ingredient soft gel capsule extract and six brands of multicomponent soft gel capsule extract were tested.

Approximately 150 mg of sample was accurately weighed and transferred to a 1.5-ml eppendorf reaction tube, 1.0 ml of deuterated methanol-d4 or 1.0 ml of deuterated chloroform-d containing 0.05% TMS was added. The mixture was mixed on a rotary mixer for 10 s and sonicated in an ultrasound bath for 30 min, followed by further mixing on a rotary mixer. The solutions were centrifuged for 5 min at 13 000 rpm. Six hundred microlitres of the supernatant was added to a 5 mm diameter NMR spectroscopy tube, and the samples were submitted for ^1^H NMR spectroscopy analysis.

### NMR spectroscopy measurements

The 1H NMR spectra were acquired using 500 MHz NMR Bruker Avance spectrometer (Bruker BioSpin GmbH, Rheinstetten, Germany) equipped with a multinuclear probe head with z-gradient. The acquisition parameters were: size of the spectra 64 k data points, line broadening factor = 0.16 Hz, pulse width = 30 degrees and the relaxation delay d1 = 1 s. TOPSPIN version 1.3 software was used for spectra acquisition and processing. The NMR was set up for the analysis applying diffusion-edited ^1^H NMRspectroscopy with suppression of ethanol and water signals. The scans were locked at zero on the TMS peak. Two hundred fifty-six scans was the number of scans chosen for optimum resolution of peaks.

### Statistical analysis

All spectra were phase-corrected, baseline-corrected, zeroed to the TMS peak and transferred to AMIX (Analysis of Mixtures software v3.0, BrukerBiospin, Rheinstetten, Germany). Before multivariate analysis, the spectra were reduced in complexity by using the ‘bucketing’ function found in AMIX to generate a number of integrated regions of the data set. The spectra in the range of 0.0–10.0 ppm were divided into 251 regions (buckets) of 0.04 ppm, and the signal intensity in each area was integrated. Water signals (4.50–5.00 ppm), residual proton signals corresponding to MeOD (3.24–3.35 ppm), CDCl_3_ (7.1–7.4 ppm) and TMS internal standard (−0.1 to 0.1 ppm) were excluded. These data were imported to Microsoft EXCEL where the samples were relabelled SP1 to SP37. The data were then imported into the SIMCA Version 13.0 for PCA analysis. The data were normalised using peak range normalisation.

## Results and Discussion

### Determination of fatty acids using GC

As these products are used in a therapeutic and disease-preventive context, it is essential to analyse the products from a perspective of daily therapeutic doses. To gain an understanding of the composition of the products in the context of their use as health-care products for the 46 products, we calculated the amount of fatty acids per day as the sum of the most prominent fatty acids in saw palmetto, lauric acid, capric acid, caprylic acid, myristic acid, palmitic acid, linolenic acid, oleic/linoleic acid and stearic acid for the lowest daily dosage as given by the manufacturer on the package. We chose the lowest daily dosage because for some products, there was a range in the dosage given, for example, two to six tablets per day, and the manufacturer of the product considers this lowest dosage still to be therapeutically beneficial.

The daily dosages were heterogenous. For 20 products, it was one unit per day, for 21 products twice and for one preparation thrice daily; for three products, there was a 4× daily and for one product even a 6× daily recommended intake. Also, the amount of saw palmetto extract declared for one tablet or capsule varied widely from 2.5 to 500 mg per unit.

**Figure 1:**
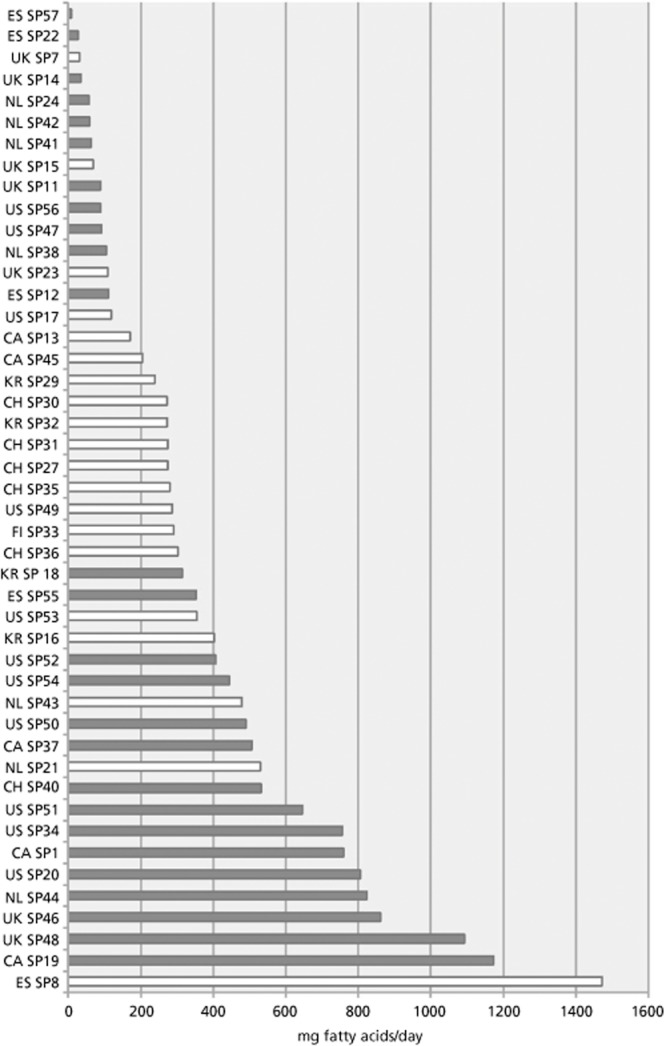
Daily dose of fatty acids based on the lowest daily dosage given on the package for each product analysed. White bars indicate monopreparations containing only saw palmetto as active constituent, dark bars combination preparations that in addition contain other ingredients like vitamins or other herbal extracts. For each product, the country and the specimen number are given.

The analysis showed that there was great heterogenicity between the products in the amount of fatty acids per day (Figure 1) for the lowest daily dosage per product. The concentrations were in the range between 8.43 mg for the product with the lowest daily amount of fatty acids and 1473 mg for the preparation with the highest value.

Mono and combination products differed slightly in the average when looking at all-test items regarding the content of fatty acids, but the combinations displayed a larger variability. Products containing only saw palmetto had an average daily dose of 230.5 ± 127.2 mg fatty acids per day (mean ± standard deviation) with values ranging from 30.89 to 1473.2 mg, combination products of 261.0 ± 247.5 mg with a range from 8.34 to 1173.02 mg daily. The corresponding median values were 271.9 mg for monopreparations and 239.3 mg/day for combinations, respectively.

The differences in the daily amount of fatty acids per product were more pronounced when looking at the country of origin. The monopreparations from Switzerland and South Korea all showed similar concentrations, while the highest dosed US American product contained 2.7 times the amount of the preparation with the lowest dose (a Spanish product); for Canada, this ratio was 3.0, and for Spain even 13.3.

For the monopreparations, we observed a large difference between the amount of saw palmetto extract as declared on the package and the measured amount of fatty acids. The differences ranged from about one-tenth fatty acids up to 4.6 more fatty acids compared with the declaration given on the package (Table 1).

**Table 1:** Characteristics of the monopreparations comparing the difference between the amount of saw palmetto extract as declared on the package and the measured concentration of total fatty acids assuming the extract would consist 100% of fatty acids

Sample number	Country	Tablet (t) or hard gel capsule (hc) or soft gel capsule (sc)	Amount of saw palmetto extract per unit (mg) as declared on package	Total fatty acids (mg) per unit measured	Difference declaration versus measurement (%)
SP8	ES	t	80	368.3	460.4
SP37	CA	sc	80	253.06	316.3
SP34	US	sc	160	378.2	236.4
SP53	US	sc	160	354.61	221.6
SP54	US	sc	320	444.31	138.8
SP36	CH	sc	160	151.56	94.7
SP33	FI	sc	320	290.24	90.7
SP49	US	sc	320	285.68	89.3
SP35	CH	sc	320	281.34	87.9
SP27	CH	sc	320	274.53	85.8
SP31	CH	sc	320	274.45	85.8
SP32	KR	sc	320	271.9	85.0
SP30	CH	sc	320	271.82	84.9
SP29	KR	sc	320	237.74	74.3
SP12	ES	hc	160	55.68	34.8
SP45	CA	hc	350	51.01	14.6
SP14	UK	hc	300	34.5	11.5
SP13	CA	hc	500	56.8	11.4
SP11	UK	hc	450*	44.57	9.9

* Also contains saw palmetto powder.

Usually lipophilic saw palmetto extracts contain 70–95% fatty acids (Table 1).

**Figure 2:**
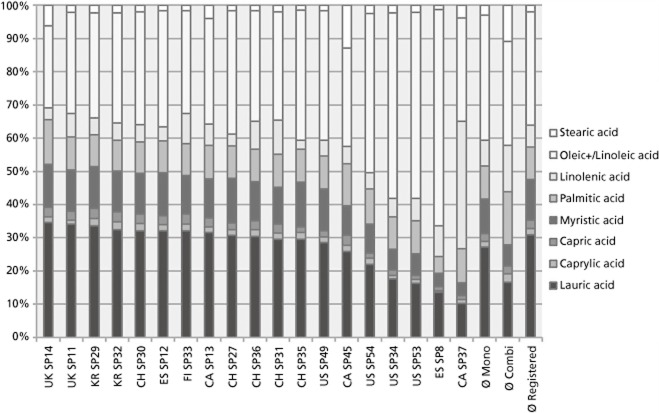
The percentage of each single fatty acid measured for each product. The bar ‘Ø registered’ shows the value of all registered products, ‘Ø mono’ the average values of all 19 monopreparations and ‘Ø combi’ the average values of all the 27 combination products. For each product, the country and the specimen number are given.

The composition of the saw palmetto mono-extracts was in the majority comparable (Figure 2). The percentage of the nine different single fatty acids was more or less comparable in about 14 of the monopreparations; the samples SP54, 34, 53, 8 and 37 differed the most from the average values.

Saw palmetto fruits contain high amounts of oleic and lauric acid; each is present with around 30–40% of the total fatty acids,[25] and the previously mentioned 14 preparations showed high concentrations of lauric and oleic/linoleic acid as well. The amount of 20% lauric acid per saw palmetto extract as demanded in the European Pharmacopeia[14] was reached by all but four monopreparations. Three of these four products, SP34, SP54 and SP8, had larger amounts of oleic and linoleic acid and one item, SP37, contained a very high concentration of linolenic acid that is unusual for saw palmetto berries and gave rise to a suspected adulteration with another plant oil. This conclusion was supported by the ^1^H NMR spectroscopy analysis (see later)

On average, the combination products showed a different pattern compared with saw palmetto preparation. They contained less lauric, oleic/linoleic and myristic acid but higher concentrations of palmitic, linolenic and stearic acid.

### ^1^H NMR spectroscopy and PCA

**Figure 3:**
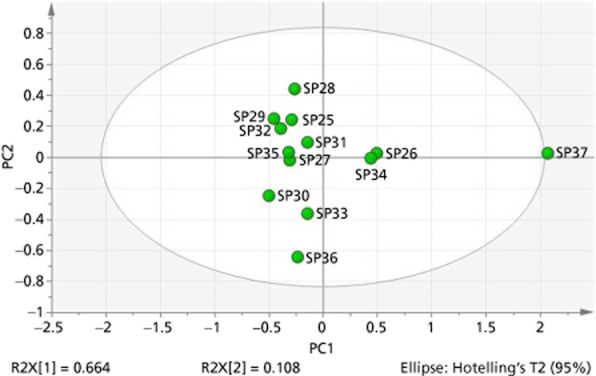
Ellipse scores plot showing soft gel extracts in chloroform-d (SP25 – SP37).

A PCA model of the ^1^H NMR spectroscopy fingerprints of the investigated products in deuterated chloroform-d showed that most clustered in one well-defined region with SP26, SP34 and SP37 being different from the main group (Scores plot, Figure 3). The scores plot is a graphical representation of the variance in chemical composition of the different products. The three extracts were yellow-orange in colour rather than the brown-green colour of the European extracts. On examination of the product specification, it was discovered that SP26 utilises super critical fluid extraction, SP34 has the added ingredients of extra virgin olive oil, glycerin, carob and zinc oxide, and SP37 has the added ingredients of soya bean oil and glycerin that could explain the differences (Figure 3).

**Figure 4:**
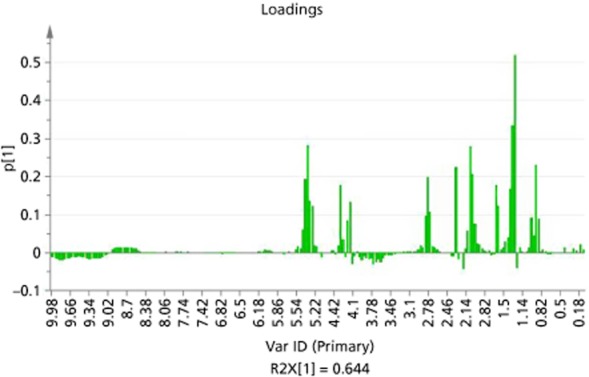
Bar chart showing loadings plot for soft gel extracts in chloroform-d (SP25 – SP37).

The loadings plot (Figure 4) shows the chemical shifts that are responsible for the main variances in the soft gel capsule extract samples. It can be seen that the major variances occur in the fatty acid regions, including oleic acid (5.3–5.5 ppm) and also in the esterified region, where caproic acid ethyl ester has been identified (from reference standards) (4.1–4.2 ppm). It is probable that the extraction methods used for these samples have increased the quantities of fatty acids, and methyl and ethyl esters in these products. The comparatively larger intensities of peaks observed in these regions may be attributable to the use of super fluid critical extraction or the addition of olive oil, soya bean oil and glycerin.

**Figure 5:**
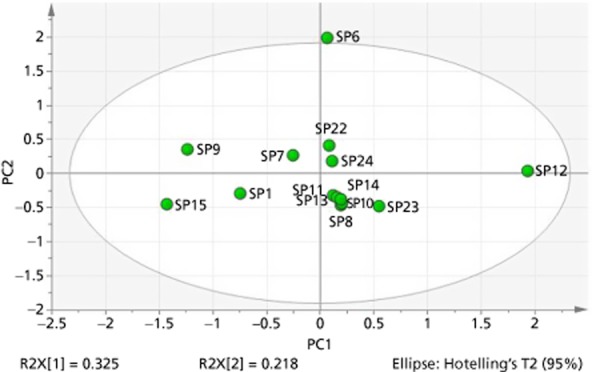
Ellipse scores plot based on NMR spectroscopy data for saw palmetto crude powders and powder extracts in methanol-d4 (SP6–SP15) versus multicombination formulae in methanol-d4 (SP1, SP22, SP23, SP24).

SP12 was shown to be the most distinctive sample (Figure 5); it was also the only one that was found to be an n-hexane, liposterolic extract with the added ingredient of polyoxethylene glycol (Figure 5). It is highly probable that the variance observed was due to a combination of these factors. Examination of the spectra of SP12, as in previous experiments, showed a very large peak between 3.62 and 3.64 ppm. This peak was absent from any other of the spectra and is suspected to be caused by the polyoxethylene glycol (from NMR spectroscopy reference data). SP6 also shows some variation with the main group, and examination of the spectra shows a marked difference in peak positions and intensities in 3.4–3.8 region, a doublet peak at 5.4 ppm, and a large singlet peak at 9.54 ppm, indicating the possible presence of an aldehyde.

**Figure 6:**
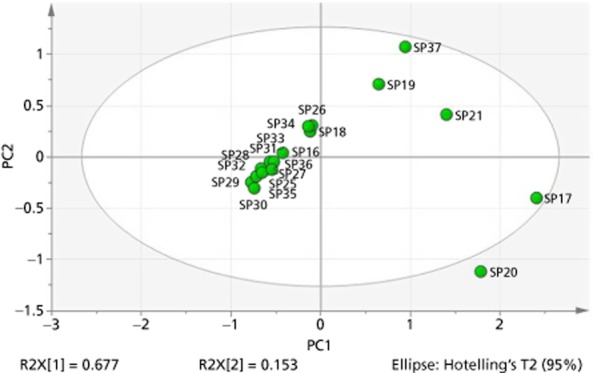
Ellipse scores plot based on NMR spectroscopy data for saw palmetto soft gel extracts (SP25–SP37) versus multicomponent formulae in chloroform-d (SP16–SP21).

It was possible to identify variances in multicomponent formulae from pure saw palmetto soft gel capsule extracts (Figure 6). The combination products were not well-grouped, but this was to be expected as they were all made up of different herbal ingredients. There was good grouping of samples of extracts to the centre and left in the scores plot, whereas all of the multicomponent formulae were placed in the right-hand quadrants. The principal axes of the scores plot represents the first and second PC scores, and so the relative variation of each component can be observed in each quadrant, e.g. scores in the upper right quadrant represent relalatively high concentrations of both PC1 and PC2.

Currently, this phytochemical analysis of 57 commercial preparations from nine different countries is the most comprehensive investigation of saw palmetto products carried out so far.

With the standard GC method, nine fatty acids were analysed, which comprise more than 95% of the fatty acids of saw palmetto berries.[25] The analysis showed they varied widely with a factor of about 177 between the minimum and maximum concentration in mono products and 38 for combinations.

In general, saw palmetto monographs recommend a daily dose of 320 mg lipophilic berry extract with 70–95% fatty acids.[6],[26] When this daily dosage of 224–304 mg fatty acids is taken as an acceptable quality standard, then only nine monopreparations were in this range. Sixteen combinations and five saw palmetto only brands displayed higher daily fatty acid values, 5 mono and 11 combination items lower quantities.

A too low daily amount of fatty acids may lead to a lack of efficacy, too high concentrations may not results in immediate health hazards as these fatty acids have a very low toxicity and higher dosages of saw palmetto extract for example were still very well-tolerated,[27] but it makes it impossible to compare these products in terms of their efficacy and evidence for their clinical effectiveness is lacking. These problems are exacerbated by inaccurate labelling of fatty acid content on six US samples. Mostly registered monopreparations medicinal products from Finland, Switzerland and Canada, and 12 combination products fulfilled only pharmacopoeial quality requirement.[14]

Mature saw palmetto fruits yield twice as much lauric acid than immature fruits; very mature fruits contain more oleic acid than lauric acid.[2],[28] The pattern from the nine ethanolic preparations showed that the majority of the manufacturers of the extracts used the same mixture of immature and mature fruits, and that they were a mixture from all regions of Florida as the fatty acid pattern varies significantly between the different regions in Florida.[25] The only samples that differed from the average fatty acid content were SP34, SP54 and SP8 that contained more oleic/linoleic acid than the other test items. This could be due to using more very mature fruits or that they were from a single cultivation where the berries contain more oleic acid. In an unpublished report, Berries from central Florida displayed a laureate to oleate ratio twice as high compared to samples from northern Florida.[28] This calls for a more systematic study of the supply of value chains from producers to consumers.[29]

Nevertheless, the GC method applied is limited as only fatty acids can be analysed. Other substances like β-sitosterol, γ-tocopherol and δ-tocopherols, or β-carotene that influence symptoms associated with BPH should also be determined to characterise the saw palmetto products better. We decided instead of analysing more substances to use a different approach for further assessment of the preparations.

^1^H NMR spectroscopy combined with PCA allows focusing on all with ^1^H NMR spectroscopy detectable constituents. In this study, a robust and economical method has been developed for the ^1^H NMR spectroscopy analysis of different dosage forms of saw palmetto products. By using multivariate analysis, it has been possible to identify major constituents, statistically group data and critically analyse the variation in composition of compounds within the products, but it is unlikely to be a full representation of all the compounds present in the fruit of *Serenoa repens*. One soft gel capsule extract product from America was manufactured using a super-critical fluid extraction process, and this study has shown that this product exhibits a significant difference in its metabolomic fingerprint compared with ethanol extracts. This study has demonstrated that it is possible to differentiate between saw palmetto samples that have been extracted using different solvents and extraction techniques; however, PCA was unable to detect small differences between extracts that were processed in similar ways and so may have limitations when detailed comparison of similar extracts is required.

One of the main problems associated with metabolomics is that the metabolome consists of a wide range of compounds at very different concentrations and with different polarities. At present, there is no single solvent capable of solubilising the whole range of chemical constituents. The choice of extraction solvent is thus limiting the view on the metabolome. It is widely accepted that a single analytical technique will not provide sufficient visualisation of the metabolome, and therefore, multiple techniques are needed for a comprehensive view. However, practical reasons can force us to choose an optimum analytical tool for metabolomic profiling. Consequently, it may be preferable to use a wide spectrum chemical analysis technique, which is rapid, reproducible and stable in time, while needing only the very basic sample preparation. ^1^H NMR spectroscopy is potentially an analytical tool that could meet these requirements.[30],[31] ^1^H NMR spectroscopy combined with multivariate or pattern recognition techniques is an ideal tool for large-scale plant metabolomics data collection, and a number of techniques have now been devised to develop NMR spectroscopy as a fingerprinting tool for the quality assessment of crude plant materials.[32] This included differentiation between various batches obtained from the same supplier, highlighting the potential to use the method for assessing whether the species’ extract variability versus the manufacturing process accounts for the variability.[33] Despite the overall relatively large number of samples included, the current study was limited by relatively low number of samples tested for each group of products, but as larger numbers of samples are added, the statistical analysis will become more robust and yield better data to cluster similar products.

The GC analysis provided a means to rapidly assess the concentration of known fatty acids within the saw palmetto products. The ^1^H NMR spectroscopy analysis enabled us to look at a wider range of chemical compounds and compare each of the products based on their total extractable metabolite content.[34] Both methods provided different information on the content of the products. By using GC analysis, it was possible to determine that some of the products contained higher amounts of some fatty acids associated with adulteration, and the ^1^H NMR spectroscopy analysis was able to confirm this. Importantly, ^1^H NMR spectroscopy analysis was able to provide some evidence that certain saw palmetto products were adulterated with compounds not detected by GC analysis (mainly other plant oils). Using PCA analysis, it has been possible to identify groupings within products based on their metabolomics profile. In the future, using this type of grouping, it will be possible to set up PCA models for saw palmetto products that could be used in product development, quality assurance and to aid in the detection of counterfeit products.

## Conclusions

This is the first study comparing 1H NMR-based metabolomics with an established and already validated method. Using PCA analysis, it has been possible to identify groupings within products based on their metabolomics profile. In the future, using this type of grouping, it will be possible to set up PCA models for saw palmetto products that could be used in product development, quality assurance and to aid in the detection of counterfeit products.

## Declarations

### Conflict of interest

The Authors declare that they have no conflicts of interest to disclose.

### Funding

This work has been part-funded with a charitable donation from A.Vogel Bioforce AG.

## Supporting Information

Table S1 Saw palmetto product information.
