# Supplementary material for: A phytochemical comparison of saw palmetto products using gas chromatography and 1H nuclear magnetic resonance spectroscopy metabolomic profiling
Source: J Pharm Pharmacol. 2014 Jan 13;66(6):811–22. doi: 10.1111/jphp.12198 (PMC4284019; doi:10.1111/jphp.12198)
Supplement: Table S1 — Saw palmetto product information. [file jphp0066-0811-SD1.doc]

| **Sample nr.**  **(SP)** | **NMR analysis** | **GC analysis** | **Country** | **Brandname** | **Producer** | **mono or combination** | **Tablet, capsule or tincture** | **Tablet or capsule weight [mg]** | **Amount of saw palmetto per unit [mg]**  **as given on package** | **Daily dosage**  **(units/d)** | **DER and extractant of saw palmetto extract** | **active constituents** | **Batch Nr.** |
| --- | --- | --- | --- | --- | --- | --- | --- | --- | --- | --- | --- | --- | --- |
| 1 | X | X | CAN | Prostate Perform | New Roots Herbal | combi | soft gel capsule | 1800 | 160 | 2x1: | 4 :1, (95% oil extract) | sterols and sterolins, SP  Pyg, rye flower pollen, vit E, PS, pau d’arco, Zn, lyc, Se, borage oil, olive oil, cranberry, vit B6, l-alanine, l-glycine, l-glutamic acid | 0691 |
| 2 | X |  | UK | Saw palmetto Mediherb |  | Mono | Tincture |  |  | 5ml | 1 :2 | SP | No data |
| 3 | X |  | UK | Saw palmetto Napier |  | Mono | Tincture |  |  |  | 1 :3 | SP | No data |
| 4 | X |  | UK | Saw palmetto Panacea |  | Mono | Tincuture |  |  |  | 1 :3 | SP | No data |
| 5 | X |  | UK | Saw palmetto Bioforce |  | Mono | Tincture |  |  | 2x20-30drps | 1:1,9 75% ethanol v/v | SP | No data |
| 6 | X |  | CAN | Palmiernain, Phytocaps, Homeocan |  | Mono | Capsule | No data | No data | 350mg | 12:1 | SP | No data |
| 7 | X | X | UK | Saw Palmetto Berry | Viridian | combi | hard capsule | 450 | 150mg SP extract, 132mg SP berry powder | 1x1 | For extract : 45-50% free fatty acids | SP extract, SP powder, bilberry extract, alfalfa, spirulina | 0492411 |
| 8 |  | X | ESP | Sereprostat | Robapharm ESP | mono | tablet | 450 | 80 | 4x1 | -- | SP | C04 |
| 9 | X |  | UK | Saw palmetto Chapmans |  | Mono | Capsule | No data | 450mg | ? | Crude powder | SP | No data |
| 11 | X | X | UK | Saw Palmetto | Holland&Barrett | mono | hard capsule | 450 | 450 | 2x2 | -- | SP | 243314-05 |
| 12 | X | X | ESP | Permixon | Pierre Favre SA | mono | hard capsule | 410 | 160 | 2x1 | 6–12:1, Hexan | SP | D01 |
| 13 | X | X | CAN | Saw Palmetto Berries | Natural Factors | mono | hard capsule | 500 | 500 | 3x1 | -- | SP | 598045 |
| 14 | X | X | UK | Saw Palmetto Berries | Solgar | mono | hard capsule | 520 | 300 | 1-3x1 | -- | SP powder extract, SP powder | 04023EN05OA |
| 15 | X | X | UK | Saw Palmetto & Pygeum Bark | Higher Nature | combi | hard capsule | 400 | 120 | 1-3x1 | 45% fatty acids | SP, urt root, rice bran | 18342 |
| 16 | X | X | Korea | CJ Nutra | CJ Nutra | combi | soft gel capsule | 750 | 500 | 1x1 | -- | SP, Zn, PS | No data |
| 17 | X | X | USA | Prostate Health | Schiff Nutrition Group Inc. | combi | hard capsule | 550 | 333.5 | 2x1 | - | vit D, Zn, Se, Cu, SP, Ca-d-gluCarate, lyc | 128152A |
| 18 | X | X | Korea | Chung Wae Pharma Corporation | Chung Wae Pharma | combi | soft gel capsule | 800 | 500 | 1x1 | -- | SP, Zn,soybean oil, tomato extract powder | No data |
| 19 | X | X | CAN | Prost - Force | Prairie Naturals | combi | soft gel capsule | 1362 | 80 | 1-2x2 | -- | vit B6, Zn, SP, Pyg, lyc, urt, PS, lecithin | 20907146 |
| 20 | (x) | X | USA | Life Extension | Quality Supplements and Vitamins Inc. | combi | soft gel capsule | 1800 | 160 | 2x1 | CO_2_, Standardized to 85-95% fatty acids | SP, flower pollen extract, boswellia serrata extract, urt, Pyg, lyc, phytosterols, enterolactone, B, rosemary | 02410110 |
| 21 | X | X | NL | Prostavit Forte | Bional | combi | soft gel capsule | 1400 | 160 | 1x1 | -- | PS oil, urt, SP, Zn, Se, yeast, tomatoes extract, vit E | 09E0404 |
| 22 | X | X | ESP | Biprostat | Dietéticos Intersa S.A. | combi | hard capsule | 365 | 365 | 2x1 | -- | SP, PS, Pyg, bearberry, Zn, vit B6 | 9HE7 |
| 23 | X | X | UK | Saw Palmetto Complex | Natures aid | combi | tablet | 1200 | 160 | 1-2x1 | 1 :10, 72mg fatty acids per tabl. | SP, PS | 211D0117 |
| 24 | X | X | NL | ProstaFleur Extra Forte | Bloem Natuurprodukten Winschoten bv | combi | hard capsule | 500 | 40 | 2-3x1 | 4 :1 | SP, PS*, Epilobium parviflorum*, b-sit, Se, Zn, vit E, lyc | 080514475 |
| 25 | X |  | GER | Prostess uno |  | Mono | Capsule | No data | 160 | 2x1 | DEV 10.1 (7,5-12,5:1) ;ethanol | SP | No data |
| 27 | X | X | SWI | Prostagutt Uno | Schwabe Pharma AG | mono | soft gel capsule | 500 | 320 | 1x1 | 10-14.3 :1, ethanol 90% m/m | SP | 1470510 |
| 28 | X |  | USA | Saw palmetto GNC USA | GNC USA | Combi | Capsule | No data | No data | 320mg | ? | SP, Vit E | No data |
| 29 | X | X | Korea | CJ Nutra Saw Palmetto | CJ Nutra | mono | soft gel capsule | 500 | 320 | 1x1 | -- | SP | No data |
| 30 | X | X | SWI | Prosta Urgenin | Max Zeller AG | mono | soft gel capsule | 480 | 320 | 1x1 | 8-9.52 :1, ethanol 90% V/V | SP | B0901685 |
| 31 | X | X | SWI | SabCaps | Vifor SA | mono | soft gel capsule | 480 | 320 | 1x1 | 9-11 :1, ethanol 96% V/V | SP | 0654760100 |
| 32 | X | X | Korea | Chong Kun Dang Health  Saw Palmetto | Chong Kun Dang Health | mono | soft gel capsule | 500 | 320 | 1x1 | -- | SP | No data |
| 33 | X | X | FIN | Curbisal | Pharbio | mono | soft gel capsule | 480 | 320 | 1x1 | 9-11 :1 | SP | 220181 |
| 34 | X | X | USA | Saw Palmetto Extract | Now Foods | mono | soft gel capsule | 600 | 160 | 1x2 | Standardized to 85-95% fatty acids | SP | 1214706 0239 |
| 35 | X | X | SWI | Prostasan | A.Vogel Bioforce | mono | soft gel capsule | 485 | 320 | 1x1 | DEV 9,0–12,0:1, ethanol 96% (V/V). | SP | L030561B |
| 36 | X | X | SWI | Prostadyn | Dr. Dünner AG | mono | soft gel capsule | 260 | 160 | 2x1 | 10-14 :1, ethanol 90% m/m | SP | E090276 |
| 37 | X | X | CAN | Saw Palmetto | Swiss Herbal Remedies Ltd | mono | soft gel capsule | 400 | 80 | 2x1 | 10:1 extract, 85%-95% fatty acids | SP | 34426 |
| 38 |  | X | NL | Prostaat | Phital | combi | tablet | 1450 | 100 | 2x1 | -- | lyc, Pyg, phytosterols, urt, SP, provit A, vit A, vit E, B1, vit B2, vit B3, vit B5, vit B6, vit B8, vit B11, vit B12, vit C, vit D3, vit K, choline, inositol, paba, lutein, b-sit, Ca, Cr, Fe, I, Na, Cu, Mg, Mn, Se, Mo, Se, Si, Zn, *grape seed, ginseng, ginkgo biloba*, green tea | 07J08C |
| 40 |  | X | SWI | Prostagutt F | Schwabe Pharma AG | combi | soft gel capsule | 675 | 160 | 2x1 | 10-14.3 :1, ethanol 90% m/m | SP, urt | 6760910 |
| 41 |  | X | NL | Prostalife | Bloem Natuurprodukten Winschoten bv | combi | hard capsule | 550 | 320 | 2-3x1 | 4 :1 | SP, urt root, *Epimedium*, *Muira puama*, b-sit, Se, Zn, vit B6, vit E, lyc | 091019829 |
| 42 |  | X | NL | Prosta Totaal | Distributie care bv | combi | tablet | 1450 | 100 | 1-2x1 | -- | lyc, Pyg, phytosterols, urt, SP, provit A, vit A, vit E, B1, vit B2, vit B3, vit B5, vit B6, vit B8, vit B11, vit B12, vit C, vit D3, vit K, choline, inositol, paba, lutein, b-sit, Ca, Cr, Fe, I, Na, Cu, Mg, Mn, Se, Mo, Se, Si, Zn, *grape seed, ginseng, ginkgo biloba,* green tea | 08B18E |
| 43 |  | X | NL | Prostalan Forte | VSM | combi | soft gel capsule | 666 | 160 | 2x1 | -- | SP, urt, saturated fatty acids | 071221 |
| 44 |  | X | NL | Prostavit | Bional | combi | soft gel capsule | 1400 | 2.5 | 1-3x1 | -- | SP, PS oil, urt, echinacea pallida, b-sit, Mg, vit E, Zn | 09I0202 |
| 45 |  | X | CAN | Saw Palmetto | Homeocan | mono | hard capsule | 350 | 350 | 4-6 | 12 :1, standardized to 25% fatty acids | SP | 0 0034 |
| 46 |  | X | UK | Prostavital | Health Aid | combi | soft gel capsule | 2000 | 30 | 1x1 | 12 :1 | vit E, vit C, vit B6, Mg, Zn, Cr, Cu, Se, lyc, l-alanine, l-glutamic acid, wheat germ oil, PS oil, lecithin, urt, SP, Pyg | 0710 |
| 47 |  | X | USA | Saw Palmetto & Pygeum Extract | Country Life | combi | soft gel capsule | 600 | 160 | 2x1 | 9 :1, standardized to 45% fatty acids=72mg/caps | SP, Pyg, plant sterol complex, soy phosphatides (soy lecithin), lipase | 10A996A |
| 48 |  | X | CAN | Saw Palmetto & Pygeum | Preferred Nutrition | combi | soft gel capsule | 1800 | 80 | 2-4x1 | 85% fatty acids | b-sit, PS oil, flower pollen extract, SP, urt, Pyg,  Zn, hydrangea root, lyc, Cu | 616880 |
| 49 |  | X | USA | ProstActive | Nature’s Way | mono | soft gel capsule | 480 | 320 | 1x1 | 12:1 | SP | 589696 |
| 50 |  | X | USA | Prostate 5LX | New Chapter Inc. | combi | soft gel capsule | 750 | 160 | 2x1 | Supercritical extract, 136mg fatty acids/caps (=85%) | SP, Se, probiotic nutrients, green tea , PS, ginger, urt, rosemary | 40503009A |
| 51 |  | X | USA | Prostate Advantage | Enzymatic Therapy Inc. | combi | soft gel capsule | 555 | 80 | 2x1 | Standardized to 85-95% fatty acids | SP, PS, Pyg | 103392 |
| 52 |  | X | USA | Saw Palmetto & Pygeum | Nature's Way | combi | soft gel capsule | 850 | 350 | 1x1 | Standardized to 85-95% fatty acids | vit B6, SP, Pyg | 589422 |
| 53  (=26) |  | X | USA | Saw Palmetto Berry Extract | Solaray | mono | soft gel capsule | 570 | 160 | 1x1-2 | 85% fatty acids and sterols | SP | 132812 |
| 54 |  | X | USA | Saw Palmetto Extract | Source Naturals | mono | soft gel capsule | 700 | 320 | 1x2 | 85-95% fatty acids and sterols | SP | 1001807 |
| 55 |  | X | ESP | Prosta - Fort | GSN Compañia General Suplementos Nutricionales | combi | tablet | 600 | 160 | 2x1 | 10 :1 | SP, urt, Pyg | B317 |
| 56 |  | X | USA | Prosta - Max | Country Life | combi | tablet | 1600 | 50 | 2x1 | -- | vit A, vit C, vit D, vit B1, vit B2, vit B6, Ca, Zn Cu glycine l-glutamic acid l-alanine plant sterol complex, b-sis, SP, Pyg, urt, lyc | 10A321A |
| 57 |  | X | ESP | Spasmo - Urgenin | Madaus | combi | tablet | 450 | 25 | 2x3 | -- | SP, *echinacea angustifolia* | Z-04 |
